# Tubular Damage Biomarkers Are a Useful Tool for Identifying Early Renal Injury in Long COVID

**DOI:** 10.3390/ijms27052420

**Published:** 2026-03-06

**Authors:** Caio V. B. Menário, Rodrigo P. Silva-Aguiar, Douglas E. Teixeira, Gabriela S. Nascimento, Nina R. G. R. Visconti, Luana S. Andrade, Fernanda C. Q. Mello, José R. Lapa-e-Silva, Nazareth N. Rocha, Camila M. Martins, Fernanda F. Cruz, Ana Acacia S. Pinheiro, Pedro L. Silva, Patricia R. M. Rocco, Celso Caruso-Neves

**Affiliations:** 1 Instituto de Biofísica Carlos Chagas Filho, Universidade Federal do Rio de Janeiro, Rio de Janeiro 21941-902, RJ, Brazil; vettorazzicaio@gmail.com (C.V.B.M.); rpachecoufrj@gmail.com (R.P.S.-A.); douglaseteixeira@gmail.com (D.E.T.); gabrielasnasufrj@gmail.com (G.S.N.); ffcruz@biof.ufrj.br (F.F.C.); acacia@biof.ufrj.br (A.A.S.P.); pedroleme@biof.ufrj.br (P.L.S.); prmrocco@biof.ufrj.br (P.R.M.R.); 2 National Institute of Advanced Technologies in Diagnosis and Prognosis of Chronic and Neglected Diseases (INATEC), Rio de Janeiro 21941-902, RJ, Brazil; ninavisconti@gmail.com (N.R.G.R.V.); pneumologia.luana@gmail.com (L.S.A.); fcqmello@idt.ufrj.br (F.C.Q.M.); jrlapa@hotmail.com (J.R.L.-e.-S.); nazarethrocha51@gmail.com (N.N.R.); camila.marinelli@aacet.com.br (C.M.M.); 3 Institute of Thoracic Diseases, Clementino Fraga Filho University Hospital (HUCFF), Universidade Federal do Rio de Janeiro, Rio de Janeiro 21941-617, RJ, Brazil; 4 Biomedical Institute, Fluminense Federal University, Rio de Janeiro 24210-201, RJ, Brazil; 5 Department of Medicine, State University of Ponta Grossa, Ponta Grossa 84030-900, PR, Brazil; 6 Network of Studies for Understanding the Pathogenesis of Late Effects of COVID-19, Carlos Chagas Filho Foundation for Research Support of the State of Rio de Janeiro (FAPERJ), Rio de Janeiro 20020-000, RJ, Brazil

**Keywords:** long COVID, post-COVID-19 syndrome, renal injury biomarkers, subclinical acute kidney injury

## Abstract

Patients without overt glomerular dysfunction may develop tubular injury, referred to as subclinical acute kidney injury. The burden of COVID-19-related renal damage may therefore be underestimated, as current KDIGO criteria do not include tubular damage biomarkers (TDBs). This study evaluated kidney injury in patients with long COVID by assessing TDBs alongside glomerular biomarkers, proteinuria (UPCr) and albuminuria (UACr). In this cross-sectional study, 75 patients without prior chronic kidney disease were recruited from a long COVID outpatient clinic and stratified according to the time since SARS-CoV-2 infection into 6-, 12-, and 24-month post-COVID-19 groups (referred to as 6-, 12-, and 24-MPC, respectively). Overall, 49.3% of patients had normal estimated glomerular filtration rate (eGFR >90 mL/min/1.73 m^2^), 34.7% showed mildly reduced eGFR (90–60), and 16% exhibited marked eGFR reduction (<60). Among patients with normal eGFR, the combined mean prevalence (mean ± SD) of abnormal TDBs, UACr, and UPCr was 29.7 ± 4.9%, indicating early tubular injury. Temporal analysis revealed a higher prevalence of TDB abnormalities at 6-MPC, whereas glomerular dysfunction was more pronounced at 24-MPC. These findings suggest that renal injury in long COVID is more prevalent than previously recognized and that TDB assessment may improve early detection of kidney damage.

## 1. Introduction

Several studies have demonstrated that a subset of patients with COVID-19 develop persistent symptoms and/or late-onset complications, a condition now recognized as long COVID [1,2,3,4,5]. It is a syndrome with a broad clinical spectrum that affects patients following acute infection with severe acute respiratory syndrome coronavirus 2 (SARS-CoV-2) [2,3,4]. Long COVID comprehends a wide range of symptoms, including fatigue, dyspnea, depression, cognitive impairment, and disturbances in taste and smell [1,3]. Among the organs affected, the kidneys have emerged as a major site of long-term injury [5,6,7,8]. The diagnosis is clinical, and symptoms of this chronic condition may develop during the acute phase of viral infection and typically persist for three months, regardless of disease severity, or may have a delayed onset, appearing weeks or even months after the acute phase. It is a diagnosis of exclusion and cannot be attributed to alternative diagnoses [2,3,4].

In a recent meta-analysis of observational studies, Zhang et al. (2023) reported that approximately 22% of COVID-19 survivors experience a sustained decline in renal function, with an increased risk of developing chronic kidney disease (CKD) [9]. The duration of follow-up after acute infection and the severity of the initial illness are two critical factors influencing long-term outcomes in long COVID [10,11]. However, the true extent of COVID-19-related renal injury may be underestimated. Patients without overt glomerular dysfunction (i.e., without elevated serum creatinine) may still exhibit tubular damage, a condition referred to as subclinical acute kidney injury (subAKI) [12,13]. These patients often go undetected using current KDIGO (Kidney Disease: Improving Global Outcomes) disease diagnostic criteria, which rely primarily on changes in serum creatinine or urine output [14].

subAKI encompasses a broad range of parenchymal renal damage, with or without subtle alterations in glomerular structure and function, even in the absence of fulfillment of KDIGO criteria. It is increasingly recognized as an emerging clinical entity and a risk factor for the subsequent development of AKI and CKD [12,15]. subAKI has been associated with adverse outcomes even among patients who do not meet diagnostic criteria for established AKI and CKD [12,16].

Several studies have reported the occurrence of subAKI during the acute phase of COVID-19 and have correlated it with adverse renal outcomes in hospitalized patients [17,18,19,20]. However, the identification and characterization of subAKI in the context of long COVID remains to be elucidated. Emerging evidence suggests that incorporating urinary tubular damage biomarkers (TDBs), such as β2-microglobulin (Uβ2M) and kidney injury molecule-1 (UKIM1), alongside measures of proteinuria and albuminuria, may improve the detection of subAKI in this population [12,21,22].

We hypothesized that tubular injury may be detectable earlier than glomerular dysfunction in patients with long COVID. To investigate this, we conducted a cross-sectional clinical study involving 75 patients previously hospitalized with mild to severe COVID-19. Participants were evaluated at 6, 12, and 24 months after their initial SARS-CoV-2 infection. Importantly, none of these patients had a prior history of kidney disease. Renal function was evaluated using the classical biomarker of glomerular function, serum creatinine, along with urinary TDBs, Uβ2M and UKIM1, and proteinuria and albuminuria.

## 2. Results

### 2.1. Demographics, Comorbidities, and Clinical Characteristics of COVID-19 Patients

The study cohort included 75 patients with a median age of 59 years, of whom 58.7% were female. At the time of hospitalization for acute COVID-19, 93.3% had at least one pre-existing comorbidity (Table 1). Hypertension (57.3%), obesity (38.6%), and diabetes mellitus (29.3%) were the most common conditions. A history of smoking was reported by 70.7% of participants, a notable risk factor for adverse COVID-19 outcomes. Baseline characteristics did not differ significantly among the post-COVID-19 follow-up groups (6-MPC, 12-MPC, and 24-MPC).

COVID-19 severity during acute illness was categorized according to World Health Organization (WHO) criteria and differed significantly among groups [23]. The 6-MPC group included a higher proportion of patients with mild disease (45.3%), whereas the 12-MPC and 24-MPC groups had more severe clinical profiles, with 66.7% and 46.7% presenting with severe disease and 20.8% and 26.7% with critical illness, respectively. Importantly, none of the patients had a prior diagnosis of chronic kidney disease (CKD), supporting the interpretation of renal changes as post-COVID-19 sequelae.

### 2.2. Pre-Existing Medication Use

Prior to SARS-CoV-2 infection, 37.3% of patients used statins, 14.7% used antiplatelet agents, and 4% used anticoagulants (Supplementary Table S1). Angiotensin-converting enzyme (ACE) inhibitors or angiotensin receptor blockers (ARBs) were used by 53.3%, while 21.3% used diuretics and 20% were prescribed beta-blockers. Regarding metabolic and endocrine therapies, 26% used oral hypoglycemics, and 11% used insulin. Additionally, 12% reported the use of vitamin D or immunosuppressants, and 6.7% used bronchodilators. No significant differences in medication usage were found among the follow-up groups, reducing potential confounding by pharmacologic exposure.

### 2.3. Vaccination Status and Respiratory Function During Acute COVID-19 Hospitalization

Vaccination status differed markedly by group: 86.1% of patients in the 6-MPC group had received at least one dose of a COVID-19 vaccine, compared with 33.3% in the 12-MPC group and just 6.7% in the 24-MPC group (Supplementary Table S2). Respiratory status during acute illness showed a mean oxygen saturation (SpO_2_) of 93.3% and a mean respiratory rate (RR) of 22.4 breaths per minute across the cohort. Patients in the 6-MPC group exhibited better respiratory parameters, while tachypnea and higher RR were more common in the 12- and 24-MPC groups, consistent with greater disease severity at presentation.

### 2.4. Inflammatory and Biochemical Profiles During Acute COVID-19 Hospitalization

Patients in the 12- and 24-MPC groups demonstrated more pronounced inflammatory responses, characterized by lymphopenia and elevated levels of CRP, D-dimer, ferritin, ALT, and LDH. In contrast, the 6-MPC group showed isolated increases in D-dimer and ferritin, without evidence of lymphopenia (Supplementary Table S2). Platelet counts and plasma creatinine remained within normal ranges during hospitalization in all groups, suggesting preserved renal filtration function during the acute phase.

### 2.5. Therapeutic Interventions and In-Hospital Adverse Events

Nearly half of the patients (46.7%) received corticosteroids during hospitalization, with substantially higher use in the 12-MPC group (95.8%), likely reflecting evolving treatment guidelines during the pandemic. Oxygen supplementation was administered to 50.7% of the cohort, predominantly in the 12- and 24-MPC groups. ICU admission rates mirrored disease severity, with 70.8% of the 12-MPC group and 26.7% of the 24-MPC group requiring intensive care. Cardiac arrhythmias and acute kidney injury (AKI) were the most frequent complications, with AKI significantly more prevalent in the 24-MPC group (Supplementary Table S3).

### 2.6. Long-Term Renal Function and Laboratory Findings

At follow-up, all groups showed normal ranges for hemoglobin, lymphocytes, and platelet counts (Supplementary Table S4). There were no statistically significant differences in inflammatory markers (CRP, D-dimer, ferritin) or indicators of tissue injury (LDH, ALT, CPK).

**Figure 1:**
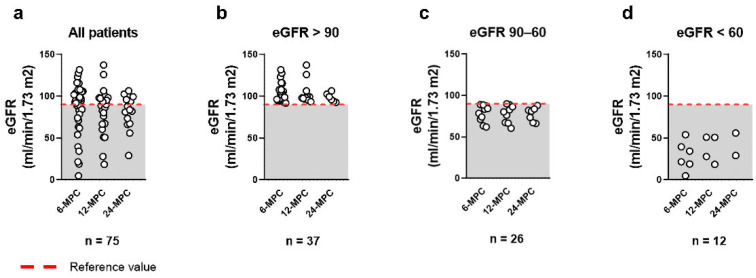
Estimated glomerular filtration rate (eGFR) was calculated from serum creatinine using the CKD-EPI equation. Patients were assessed at the following time points: (1) 6 months post-COVID-19 (6-MPC; *n* = 36), (2) 12 months post-COVID-19 (12-MPC; *n* = 24), and (3) 24 months post-COVID-19 (24-MPC; *n* = 15). The red dashed line indicates the reference value. (**a**) Estimated glomerular filtration rate in all patients (*n* = 75), (**b**) patients with normal eGFR (>90 mL/min/1.73 m^2^; *n* = 37), (**c**) patients with mild eGFR decline (90–60 mL/min/1.73 m^2^; *n* = 26), and (**d**) patients with marked eGFR decline (<60 mL/min/1.73 m^2^; *n* = 12).

Renal function, assessed via estimated glomerular filtration rate (eGFR), revealed that 49.3% of patients maintained normal values, 34.7% had mild eGFR decline (90–60 mL/min/1.73 m^2^), and 16% exhibited marked decline (<60 mL/min/1.73 m^2^; Figure 1).

### 2.7. Long-Term Tubular Damage Biomarkers

**Figure 2:**
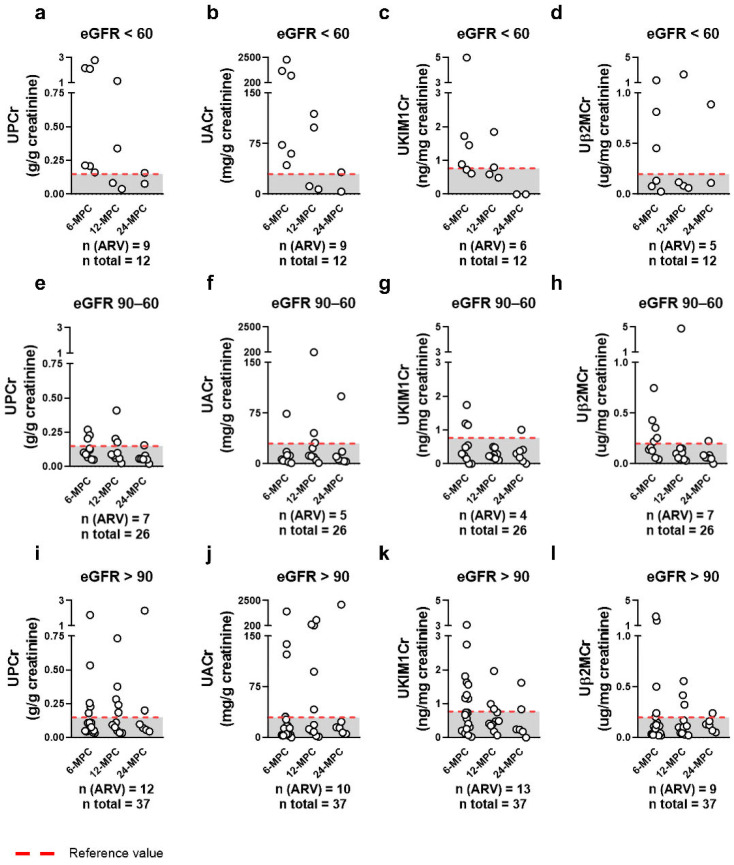
Assessment of renal injury biomarkers in patients with long COVID and (**a**–**d**) markedly reduced glomerular function (eGFR <60 mL/min/1.73 m^2^), (**e**–**h**) mildly reduced glomerular function (eGFR 90–60 mL/min/1.73 m^2^), and (**i**–**l**) normal eGFR (>90 mL/min/1.73 m^2^). The red dashed line indicates the reference value. Glomerular biomarkers (UPCr and UACr) and tubular damage biomarkers (TDBs: Uβ2MCr and UKIM1Cr) were assessed. UPCr: urinary protein-to-creatinine ratio; UACr: urinary albumin-to-creatinine ratio; UKIM1Cr: urinary kidney injury molecule-1-to-creatinine ratio; and Uβ2MCr: urinary β2-microglobulin-to-creatinine ratio. ARV: above the reference value. The prevalence was calculated considering all groups (6-MPC, 12-MPC, and 24-MPC) for each parameter using the following equation: {[n(ARV)/n total] × 100}. Median and mean values were calculated based on the prevalences of all parameters.

**Figure 3:**
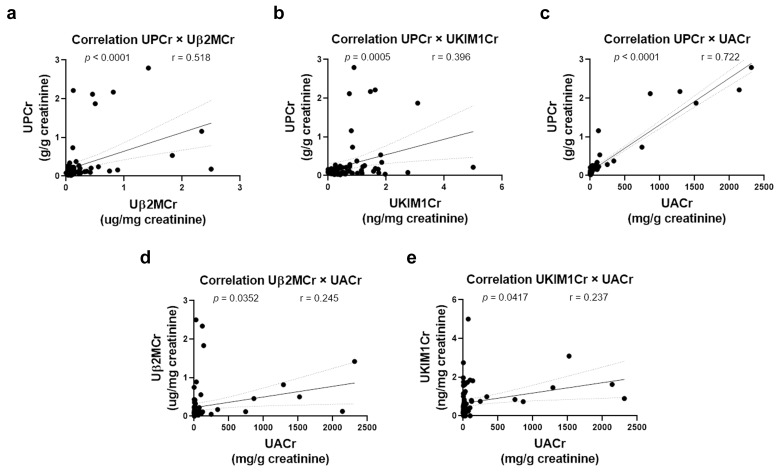
Correlation between tubular damage biomarkers (TDBs: Uβ2MCr and UKIM1Cr) and urinary protein-to-creatinine ratio (UPCr) or urinary albumin-to-creatinine ratio (UACr). Correlation analyses were performed between: (**a**) UPCr and Uβ2MCr, (**b**) UPCr and UKIM1Cr, (**c**) UPCr and UACr, (**d**) Uβ2MCr and UACr, and (**e**) UKIM1Cr and UACr. Spearman’s correlation coefficient (r) was used to analyze the relationship between variables. Statistical significance was considered at *p* < 0.05. Uβ2MCr: urinary β2-microglobulin-to-creatinine ratio; UKIM1Cr: urinary kidney injury molecule-1-to-creatinine ratio.

To detect possible renal injury, we evaluated urinary protein-to-creatinine ratio (UPCr) and urinary albumin-to-creatinine ratio (UACr) associated with tubular damage biomarkers (TDBs), urinary kidney injury molecule-1-to-creatinine ratio (UKIM1Cr), and urinary β2-microglobulin-to-creatinine ratio (Uβ2MCr). The values were stratified by eGFR status (Figure 2). The median (Q1–Q3 interquartile range) and mean (mean ± SD) of prevalence of the abnormal levels of UPCr, UACr and TDBs were significantly highest, 62.5% (45.8–75.0%) and 60.4% ± 17.1% (*p* < 0.05), among patients exhibiting a marked eGFR decline (Figure 2a–d). In patients with mild eGFR decline, these values were 23.1% (17.3–26.9%) and 22.1 ± 5.7%, respectively (Figure 2e–h). Notably, patients with normal eGFR presented 29.7% (25.7–33.8%) and 29.7 ± 4.9%, respectively, suggesting early tubular injury in the absence of overt glomerular dysfunction (Figure 2i–l). The values between patients with normal and mild decline in eGFR were not statistically significant (*p* = 0.407). TDBs showed positive correlations with both UPCr and UACr, supporting their utility as early indicators of renal injury (Figure 3a–e).

### 2.8. Temporal Trends in Renal Injury

**Figure 4:**
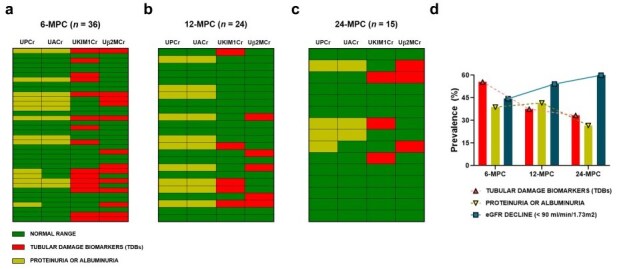
Prevalence and distribution of renal damage biomarkers and decline in glomerular function over time. (**a**–**c**) Heatmaps showing the frequency of renal damage biomarkers during the different follow-up periods: (**a**) 6 months post-COVID-19 (6-MPC), (**b**) 12 months post-COVID-19 (12-MPC), and (**c**) 24 months post-COVID-19 (24-MPC). The columns represent each biomarker (UPCr, UACr, Uβ2MCr, or UKIM1Cr), and the rows correspond to each patient. Green indicates values within normal limits. Red and yellow indicate values above the reference limits for tubular damage biomarkers (TDBs) and UPCr or UACr, respectively. (**d**) Prevalence (%) of TDBs (red bars), UPCr or UACr (yellow bars), and estimated glomerular filtration rate (eGFR) decline (<90 mL/min/1.73 m^2^, blue bars). Patients were considered positive for UPCr or UACr if at least one of these biomarkers exceeded the reference values. Patients with an abnormality in at least one tubular damage biomarker, Uβ2MCr or UKIM1Cr, were also considered positive. UPCr: urinary protein-to-creatinine ratio; UACr: urinary albumin-to-creatinine ratio; UKIM1Cr: urinary kidney injury molecule-1-to-creatinine ratio; Uβ2MCr: urinary β2-microglobulin-to-creatinine ratio.

Analysis of biomarker dynamics over time revealed a dissociation between tubular damage and glomerular decline (Figure 4a–d). Among patients with at least one elevated biomarker, the prevalence of TDB was greatest at 6 months post-infection, while reductions in eGFR were more frequent at 24 months. This temporal lag suggests that tubular damage may precede, and possibly predict, long-term decline in glomerular filtration. Heatmap visualizations confirmed higher expression of renal injury biomarkers at earlier follow-up points, reinforcing the need for early post-COVID-19 renal surveillance.

## 3. Discussion

Besides the significant socio-economic consequences during the pandemic, COVID-19 remains a challenge due to its long-term impact on various organs [24]. This process directly affects the global population, potentially leading to an increased prevalence of chronic diseases associated with high mortality and morbidity [25]. SARS-CoV-2 infection has been associated with a 35% risk of developing kidney disease [26], and long COVID increases the risk of mortality and the development of CKD [27]. In the present cross-sectional clinical study using 75 hospitalized patients, we showed the differential impact of long COVID on renal function measured by glomerular and tubular kidney damage biomarkers.

In the present manuscript, we conducted a cross-sectional study, which enables the identification of biomarkers at a specific point in time. The results obtained from this cross-sectional analysis indicate that urinary tubular damage biomarkers can be detected earlier than the glomerular dysfunction biomarkers routinely used in clinical practice. Nevertheless, our findings do not allow us to conclude that tubular damage precedes glomerular dysfunction, as could be concluded in a longitudinal cohort study.

COVID-19 severity was classified according to the NIH criteria, as detailed in the Supplementary Methods [23]. Although mild COVID-19 typically does not require hospitalization, our study population was recruited from a university hospital at different phases of the pandemic, during which some patients were admitted for clinical monitoring and management of comorbidities.

The possibility that pre-existing comorbidities influenced the results obtained can be ruled out, as the prevalence of these conditions did not differ between groups. Moreover, no patient had a prior diagnosis of CKD, and no significant differences were observed in inflammatory and biochemical markers at the time of urine collection. Additionally, a higher proportion of patients in the 6-MPC group had received more than one dose of the vaccine compared to those in the 12-MPC and 24-MPC groups. However, our data do not allow us to isolate the specific contribution of vaccination to the renal damage observed in this study.

The heterogeneity among the groups regarding the severity of acute COVID-19 may limit causal or temporal interpretations of the findings, as differences between groups with distinct clinical presentations during the acute phase could be confounded with disease progression over time. However, given the cross-sectional design of the study, neither causal relationships nor longitudinal dynamics were inferred.

Consistent with previous reports using serum creatinine-based estimations of glomerular filtration rate (eGFR) [9,28,29], our data revealed a progressive increase in the prevalence of eGFR decline (<90 mL/min/1.73 m^2^) from 6 to 24 months post-infection. This trend suggests a subtle yet potentially progressive decline in glomerular function, particularly in individuals who experienced moderate-to-severe forms of COVID-19. The association between more severe acute illness and lower eGFR values at later time points supports this interpretation. Prior cohort studies have similarly shown that eGFR decline is more pronounced among patients who required hospitalization or intensive care compared to those with milder disease [28,29].

Acute kidney injury (AKI) is a well-established predictor of poor outcomes in COVID-19 patients [30,31]. A retrospective multi-center observational cohort study showed that COVID-19 patients that develop AKI presented long-term worse impairment in kidney function [32]. In our cohort, the incidence of AKI was highest in the 24-MPC group (20%; 3 of 15 patients had a history of AKI), compared with 0% and 4.2% in the 6-MPC and 12-MPC groups, respectively. This finding may suggest an association with a decline in eGFR over time. Nevertheless, it is unlikely that the three patients with a history of AKI fully account for the differences in eGFR observed among the groups, which represents a limitation of the present study. While the limited sample size precludes firm conclusions, these findings are consistent with earlier large-scale studies indicating that AKI during the acute phase of infection is associated with long-term impairment of kidney function [32]. Further research involving larger cohorts and longitudinal follow-up is necessary to clarify the causal relationship between COVID-19–associated AKI and subsequent kidney function decline.

According to KDIGO guidelines, AKI and CKD are typically diagnosed based on elevations in serum creatinine [14,33]. However, this marker is known to be insensitive to early renal injury, as significant increases in serum creatinine usually occur only after more than 50% of nephron function is lost due to renal functional reserve [20]. This limitation underscores the importance of employing additional biomarkers that can detect subclinical or early-stage kidney damage, particularly in individuals recovering from COVID-19.

Interestingly, while eGFR decline appeared to progress over time, urinary tubular damage biomarkers, UKIM1 and Uβ2M, were most elevated in the early post-infection period (6-MPC group), with a reduction in prevalence observed at 12- and 24-MPC. This inverse temporal relationship suggests that tubular injury may occur earlier and resolve with time, whereas glomerular dysfunction may develop later as a secondary event. These observations align with reports of subAKI in COVID-19 patients, in which proximal tubular epithelial cell (PTEC) dysfunction may precede overt changes in glomerular filtration [34,35,36]. It was also observed that patients in the 6-MPC group, who experienced less severe COVID-19, exhibited a higher prevalence of tubular damage biomarkers, suggesting that the severity of acute COVID-19 may be dissociated from renal damage in long COVID. Furthermore, Werion et al. (2020) [36] demonstrated that proximal tubule dysfunction during the acute phase of COVID-19 occurred independently of disease severity, viral load, comorbidities, and the use of medications with potential tubular toxicity.

SARS-CoV-2 has been shown to directly infect PTECs [36,37], and experimental evidence suggests that the viral spike protein disrupts megalin-mediated albumin endocytosis in these cells via Toll-like receptor 4 signaling [38,39]. In addition, post-mortem studies have reported reduced megalin expression and acute tubular injury in COVID-19 patients despite preserved eGFR [36]. In our cohort, the observed association between tubular injury biomarkers and the presence of proteinuria and albuminuria further supports the role of early tubular dysfunction in impairing protein reabsorption, potentially preceding glomerular involvement.

## 4. Materials and Methods

### 4.1. Study Design

This was a cross-sectional clinical study conducted at the Long COVID Outpatient Clinic of the Clementino Fraga Filho University Hospital (HUCFF), Federal University of Rio de Janeiro (UFRJ), between March 2020 and May 2022. The study was approved by the HUCFF Research Ethics Committee (CAAE: 53517521.6.0000.5257) and was conducted in accordance with the ethical standards of the Declaration of Helsinki. The Strengthening the Reporting of Observational Studies in Epidemiology (STROBE) guidelines were followed [40]. Written informed consent was obtained from all participants prior to their inclusion.

### 4.2. Participants and Eligibility Criteria

Supplementary Table S5 shows the patient flow diagram. Patients were eligible if they met the following inclusion criteria: 1—hospitalization due to confirmed SARS-CoV-2 infection, either in a ward or intensive care unit (ICU); 2—diagnosis based on a positive RT-PCR test and/or chest computed tomography consistent with viral pneumonia; 3—age > 18 years; and 4—provision of written informed consent. Exclusion criteria were: 1—previous diagnosis of chronic kidney disease (CKD); 2—pregnancy; or 3—breastfeeding. A total of 75 patients were enrolled and stratified into three groups based on the time elapsed since their acute COVID-19 infection: (1) Group 1: 6 months post-COVID-19 (6-MPC; *n* = 36 patients); (2) Group 2: 12 months post-COVID-19 (12-MPC; *n* = 24 patients); and (3) Group 3: 24 months post-COVID-19 (24-MPC; *n* = 15 patients).

No sample size calculation was performed. We acknowledge the limitations imposed by the sample size, particularly after stratification into subgroups. The subgroup analyses should therefore be interpreted as exploratory.

### 4.3. Recruitment and Data Collection

Eligible patients were identified from hospital records and contacted by telephone. During the call, the study objectives and procedures were explained, and any questions were addressed. Patients who agreed to participate were scheduled for an in-person consultation at the Long COVID Outpatient Clinic (HUCFF). Data on the main in-hospital complications during admission, including AKI, were retrospectively obtained from hospital medical records. Patients who developed AKI during hospitalization were classified as stage 1 according to the KDIGO criteria [14]. Further details of the recruitment protocol are provided in the Supplementary Materials.

### 4.4. Urine Sample Collection and Biomarker Analysis

First-morning spot urine samples (collected between 7:00 and 9:00 a.m.) were obtained to minimize variability and reflect basal renal function. Samples were processed immediately after collection: clarified by centrifugation (10,000 × *g* for 10 min, repeated five times) and analyzed fresh to avoid degradation. The following urinary biomarkers were measured: urinary creatinine, total urinary protein, urinary albumin, urinary β2-microglobulin (Uβ2M), and urinary kidney injury molecule-1 (UKIM1). Biomarker concentrations were normalized to urinary creatinine to adjust for urine dilution. All assays and analytic procedures are detailed in the Supplementary Materials.

### 4.5. Estimation of Glomerular Filtration Rate (eGFR)

Glomerular filtration rate was estimated using the Chronic Kidney Disease Epidemiology Collaboration (CKD-EPI) equation [41]. Based on the 2024 KDIGO guidelines [33], patients were classified into the following eGFR categories: normal: >90 mL/min/1.73 m^2^; mild reduction: 90–60 mL/min/1.73 m^2^; and marked reduction: <60 mL/min/1.73 m^2^.

### 4.6. Definition of Abnormal Biomarker Levels

Reference thresholds used to classify abnormal biomarker levels were (1) urinary albumin-to-creatinine ratio (UACr): >30 mg/g [33]; (2) urinary protein-to-creatinine ratio (UPCr): >0.15 g/g [42]; (3) urinary β2-microglobulin-to-creatinine ratio (Uβ2MCr): >0.2 µg/mg [43]; (4) urinary kidney injury molecule-1-to-creatinine ratio (UKIM1Cr): >0.77 ng/mg [44,45]. Patients were classified as non-injury if all biomarkers were within reference limits. If any TDB exceeded its respective cutoff, the patient was categorized as suggestive of tubular injury. The cut-off values were not adjusted for age or sex.

### 4.7. Statistical Analysis

Due to the exploratory nature of the study, no formal sample size calculation was performed. Descriptive statistics were presented as means ± standard deviations or medians with interquartile ranges (25th–75th percentiles), depending on data distribution. Categorical variables were reported as absolute and relative frequencies.

The Shapiro–Wilk test was used to assess the normality of continuous variables. For variables without normal distribution, differences between 3 or more groups were verified using the Kruskal–Wallis test followed by the post hoc Dunn’s all-pairs rank comparison test with Bonferroni adjustment. Associations between categorical variables were analyzed using the chi-square test. Correlations between continuous variables were assessed using Spearman’s rank correlation coefficient. Scatter plots were created to evaluate the relationship between quantitative variables, and the Spearman correlation (r) was calculated. All analyses were conducted using R 4.1.0 software (R Core Team, Vienna, Austria) and GraphPad Prism 8 (GraphPad Software, San Diego, CA, USA) for graphical representation and statistical summaries. A *p*-value of < 0.05 was considered statistically significant.

## 5. Conclusions

Our findings suggest that tubular damage may be an early and potentially reversible manifestation of COVID-19-related kidney damage, while glomerular dysfunction may develop later and reflect more sustained or progressive injury. This temporal dissociation underscores the clinical value of urinary biomarkers such as KIM-1 and β2-microglobulin, along with proteinuria and albuminuria, in the early identification of subAKI in long COVID. Early detection of this syndrome is extremely important, considering that patients with subAKI have worse renal outcomes, as they are at increased risk of developing AKI and CKD [20,46,47].

## Figures and Tables

**Table 1:** Demographics, comorbidities, and clinical presentation of COVID-19 patients: Descriptive statistics and comparisons between groups.

	No	All Patients	6 Months Post COVID-19 (6-MPC)	12 Months Post COVID-19 (12-MPC)	24 Months Post COVID-19 (24-MPC)	*p*-Value Between Groups *
**Absolute and relative frequencies, *n* (%)**		**75**	**36 (48%)**	**24 (32%)**	**15 (20%)**	
**Age, MD [Q1–Q3], y**	74	59 [48–67]	60 [46–72]	56 [48–64]	63 [53–66]	0.661
**Sex, *n* (%)**	75					0.677
Male		31 (41.3%)	13 (36.1%)	11 (45.8%)	7 (46.7%)	
Female		44 (58.7%)	23 (63.9%)	13 (54.2%)	8 (53.3%)	
**Comorbidities, *n* (%)**	75	70 (93.3%)	33 (91.7%)	23 (95.8%)	14 (93.3%)	0.849
Hypertension	75	42 (57.3%)	16 (44.4%)	16 (66.7%)	10 (66.7%)	0.153
Diabetes mellitus	75	22 (29.3%)	8 (22.2%)	9 (37.5%)	5 (33.3%)	0.414
Obesity (BMI > 30)	73	29 (38.7%)	11 (32.4%)	10 (41.7%)	8 (53.3%)	0.374
Obstructive disease	75	9 (12%)	3 (8.3%)	5 (20.8%)	1 (6.7%)	0.268
Heart diseases	75	13 (17.3%)	6 (16.7%)	4 (16.7%)	3 (20%)	1
Interstitial lung disease	75	3 (4%)	1 (2.8%)	2 (8.3%)	0 (0%)	0.584
Neoplasia	75	15 (20%)	11 (30.6%)	1 (4.2%)	3 (20%)	0.034
**Smokers, *n* (%)**	75					0.107
Yes		53 (70.7%)	27 (75%)	19 (79.2%)	7 (46.7%)	
No		5 (6.7%)	3 (8.3%)	0 (0%)	2 (13.3%)	
**Clinical spectrum of COVID-19, *n* (%)**	75					<0.001
Mild		34 (45.3%)	34 (94.4%)	0 (0.0%)	0 (0.0%)	
Moderate		8 (10.7%)	1 (2.8%)	3 (12.5%)	4 (26.7%)	
Severe		24 (32%)	1 (2.8%)	16 (66.7%)	7 (46.7%)	
Critical		9 (12%)	0 (0%)	5 (20.8%)	4 (26.7%)	

The descriptive analysis of the data is presented through absolute frequencies (*n*) and percentages according to group. No = Number of observations; MD = Median; Q1 = 1st quartile; Q3 = 3rd quartile; * Statistical significance was determined by the tests described in the Section 4.7. *p* < 0.05 was considered statistically significant.

## Data Availability

The original contributions presented in this study are included in the article/Supplementary Material. Further inquiries can be directed to the corresponding author.

## Supplementary Materials

The following supporting information can be downloaded at: https://www.mdpi.com/article/10.3390/ijms27052420/s1.

## References

[1] O’Mahoney L.L., Routen A., Gillies C., Jenkins S.A., Almaqhawi A., Ayoubkhani D., Banerjee A., Brightling C., Calvert M., Cassambai S. (2025). The risk of Long COVID symptoms: A systematic review and meta-analysis of controlled studies. Nat. Commun..

[2] Skevaki C., Moschopoulos C.D., Fragkou P.C., Grote K., Schieffer E., Schieffer B. (2025). Long COVID: Pathophysiology, current concepts, and future directions. J. Allergy Clin. Immunol..

[3] Li J., Zhou Y., Ma J., Zhang Q., Shao J., Liang S., Yu Y., Li W., Wang C. (2023). The long-term health outcomes, pathophysiological mechanisms and multidisciplinary management of long COVID. Signal Transduct. Target. Ther..

[4] World Health Organization Post COVID-19 Condition (Long COVID). https://www.who.int/news-room/fact-sheets/detail/post-covid-19-condition-(long-covid).

[5] Davis H.E., McCorkell L., Vogel J.M., Topol E.J. (2023). Long COVID: Major findings, mechanisms and recommendations. Nat. Rev. Microbiol..

[6] Lineburg K.E., Smith C. (2023). The persistence of SARS-CoV-2 and its role in long COVID. NEJM Evid..

[7] Cheng Y., Luo R., Wang K., Zhang M., Wang Z., Dong L., Li J., Yao Y., Ge S., Xu G. (2020). Kidney disease is associated with in-hospital death of patients with COVID-19. Kidney Int..

[8] Legrand M., Bell S., Forni L., Joannidis M., Koyner J.L., Liu K., Cantaluppi V. (2021). Pathophysiology of COVID-19-associated acute kidney injury. Nat. Rev. Nephrol..

[9] Zhang Y., Zhao Y., Wang J., Zheng X., Xu D., Lv J., Yang L. (2023). Long-term renal outcomes of patients with COVID-19: A meta-analysis of observational studies. J. Nephrol..

[10] Huang L., Li X., Gu X., Zhang H., Ren L., Guo L., Liu M., Wang Y., Cui D., Wang Y. (2022). Health outcomes in people 2 years after surviving hospitalisation with COVID-19: A longitudinal cohort study. Lancet Respir. Med..

[11] Bowe B., Xie Y., Xu E., Al-Aly Z. (2021). Kidney Outcomes in Long COVID. J. Am. Soc. Nephrol..

[12] Haase M., Kellum J.A., Ronco C. (2012). Subclinical AKI—An emerging syndrome with important consequences. Nat. Rev. Nephrol..

[13] Strauß C., Booke H., Forni L., Zarbock A. (2024). Biomarkers of acute kidney injury: From discovery to the future of clinical practice. J. Clin. Anesth..

[14] Khwaja A. (2012). KDIGO clinical practice guidelines for acute kidney injury. Nephron Clin. Pract..

[15] Vanmassenhove J., Van Biesen W., Vanholder R., Lameire N. (2019). Subclinical AKI: Ready for primetime in clinical practice?. J. Nephrol..

[16] Fang F., Hu X., Dai X., Wang S., Bai Z., Chen J., Pan J., Li X., Wang J., Li Y. (2018). Subclinical acute kidney injury is associated with adverse outcomes in critically ill neonates and children. Crit. Care.

[17] Yasar E., Ozger H.S., Yeter H.H., Yildirim C., Osmanov Z., Cetin T.E., Akcay O.F., Bukan N., Derici U. (2022). Could urinary kidney injury molecule-1 be a good marker in subclinical acute kidney injury in mild to moderate COVID-19 infection?. Int. Urol. Nephrol..

[18] Saygili S., Canpolat N., Cicek R.Y., Agbas A., Yilmaz E.K., Sakalli A.A.K., Aygun D., Akkoc G., Demirbas K.C., Konukoglu D. (2023). Clinical and subclinical acute kidney injury in children with mild-to-moderate COVID-19. Pediatr. Res..

[19] Menez S., Moledina D.G., Thiessen-Philbrook H., Wilson F.P., Obeid W., Simonov M., Yamamoto Y., Corona-Villalobos C.P., Chang C., Garibaldi B.T. (2022). Prognostic Significance of Urinary Biomarkers in Patients Hospitalized With COVID-19. Am. J. Kidney Dis..

[20] Silva-Aguiar R.P., Teixeira D.E., Peres R.A.S., Peruchetti D.B., Gomes C.P., Schmaier A.H., Rocco P.R.M., Pinheiro A.A.S., Caruso-Neves C. (2022). Subclinical acute kidney injury in COVID-19: Possible mechanisms and future perspectives. Int. J. Mol. Sci..

[21] Ronco C., Kellum J.A., Haase M. (2012). Subclinical AKI is still AKI. Crit. Care.

[22] Lin K.M., Su C.C., Chen J.Y., Pan S.Y., Chuang M.H., Lin C.J., Wu C.J., Pan H.C., Wu V.C. (2024). Biomarkers in pursuit of precision medicine for acute kidney injury: Hard to get rid of customs. Kidney Res. Clin. Pract..

[23] National Institutes of Health (US) COVID-19 Treatment Guidelines Panel. Coronavirus Disease 2019 (COVID-19) Treatment Guidelines. https://www.ncbi.nlm.nih.gov/books/NBK570371/pdf/Bookshelf_NBK570371.pdf.

[24] Al-Aly Z., Davis H., McCorkell L., Soares L., Wulf-Hanson S., Iwasaki A., Topol E.J. (2024). Long COVID science, research and policy. Nat. Med..

[25] Nalbandian A., Sehgal K., Gupta A., Madhavan M.V., McGroder C., Stevens J.S., Cook J.R., Nordvig A.S., Shalev D., Sehrawat T.S. (2021). Post-acute COVID-19 syndrome. Nat. Med..

[26] Pan B., Wang X., Lai H., Vernooij R.W.M., Deng X., Ma N., Li D., Huang J., Zhao W., Ning J. (2024). Risk of kidney and liver diseases after COVID-19 infection: A systematic review and meta-analysis. Rev. Med. Virol..

[27] Zhang Y., Ba D.M., Risher K., Liao D., Parent L.J., Ghahramani N., Chinchilli V.M. (2024). Effects of ACE inhibitor/ARB therapy and long COVID on kidney disease: A retrospective cohort study using real-world data. Clin. Kidney J..

[28] Mahalingasivam V., Faucon A.L., Sjölander A., Bosi A., González-Ortiz A., Lando S., Fu E.L., Nitsch D., Bruchfeld A., Evans M. (2024). Kidney Function Decline After COVID-19 Infection. JAMA Netw. Open.

[29] Bowe B., Cai M., Xie Y., Gibson A.K., Maddukuri G., Al-Aly Z. (2020). Acute Kidney Injury in a National Cohort of Hospitalized US Veterans with COVID-19. Clin. J. Am. Soc. Nephrol..

[30] Kolhe N.V., Fluck R.J., Selby N.M., Taal M.W. (2020). Acute kidney injury associated with COVID-19: A retrospective cohort study. PLoS Med..

[31] Zahid U., Ramachandran P., Spitalewitz S., Alasadi L., Chakraborti A., Azhar M., Mikhalina G., Sherazi A., Narh J.T., Khattar P. (2020). Acute Kidney Injury in COVID-19 Patients: An Inner City Hospital Experience and Policy Implications. Am. J. Nephrol..

[32] Tan B.W., Tan B.W., Tan A.L., Schriver E.R., Gutiérrez-Sacristán A., Das P., Yuan W., Hutch M.R., García Barrio N., Pedrera Jimenez M. (2022). Long-term kidney function recovery and mortality after COVID-19-associated acute kidney injury: An international multi-centre observational cohort study. EClinicalMedicine.

[33] (2024). Kidney Disease: Improving Global Outcomes (KDIGO) CKD Work Group. KDIGO 2024 Clinical Practice Guideline for the Evaluation and Management of Chronic Kidney Disease. Kidney Int..

[34] Sun D.Q., Wang T.Y., Zheng K.I., Targher G., Byrne C.D., Chen Y.P., Zheng M.H. (2020). Subclinical Acute Kidney Injury in COVID-19 Patients: A Retrospective Cohort Study. Nephron.

[35] Kormann R., Jacquot A., Alla A., Corbel A., Koszutski M., Voirin P., Garcia Parrilla M., Bevilacqua S., Schvoerer E., Gueant J.L. (2020). Coronavirus disease 2019: Acute Fanconi syndrome precedes acute kidney injury. Clin. Kidney J..

[36] Werion A., Belkhir L., Perrot M., Schmit G., Aydin S., Chen Z., Penaloza A., De Greef J., Yildiz H., Pothen L. (2020). SARS-CoV-2 causes a specific dysfunction of the kidney proximal tubule. Kidney Int..

[37] Su H., Yang M., Wan C., Yi L.X., Tang F., Zhu H.Y., Yi F., Yang H.C., Fogo A.B., Nie X. (2020). Renal histopathological analysis of 26 postmortem findings of patients with COVID-19 in China. Kidney Int..

[38] Silva-Aguiar R.P., Teixeira D.E., Peruchetti D.B., Florentino L.S., Peres R.A.S., Gomes C.P., Marzolo M.P., Rocco P.M.R., Pinheiro A.A.S., Caruso-Neves C. (2022). SARS-CoV-2 spike protein inhibits megalin-mediated albumin endocytosis in proximal tubule epithelial cells. Biochim. Biophys. Acta Mol. Basis Dis..

[39] Silva-Aguiar R.P., Teixeira D.E., Peruchetti D.B., Peres R.A.S., Alves S.A.S., Calil P.T., Arruda L.B., Costa L.J., Silva P.L., Schmaier A.H. (2024). Toll like receptor 4 mediates the inhibitory effect of SARS-CoV-2 spike protein on proximal tubule albumin endocytosis. Biochim. Biophys. Acta Mol. Basis Dis..

[40] Vandenbroucke J.P., von Elm E., Altman D.G., Gøtzsche P.C., Mulrow C.D., Pocock S.J., Poole C., Schlesselman J.J., Egger M., STROBE Initiative (2014). Strengthening the Reporting of Observational Studies in Epidemiology (STROBE): Explanation and elaboration. Int. J. Surg..

[41] Levey A.S., Stevens L.A., Schmid C.H., Zhang Y.L., Castro A.F., Feldman H.I., Kusek J.W., Eggers P., Van Lente F., Greene T. (2009). A new equation to estimate glomerular filtration rate. Ann. Intern. Med..

[42] Nakamura A., Miyoshi H., Kameda H., Yamashita K., Kurihara Y. (2020). Impact of sodium-glucose cotransporter 2 inhibitors on renal function in participants with type 2 diabetes and chronic kidney disease with normoalbuminuria. Diabetol. Metab. Syndr..

[43] Hettinga Y.M., Scheerlinck L.M., Lilien M.R., Rothova A., de Boer J.H. (2015). The value of measuring urinary β2-microglobulin and serum creatinine for detecting tubulointerstitial nephritis and uveitis syndrome in young patients with uveitis. JAMA Ophthalmol..

[44] Han W.K., Bailly V., Abichandani R., Thadhani R., Bonventre J.V. (2002). Kidney Injury Molecule-1 (KIM-1): A novel biomarker for human renal proximal tubule injury. Kidney Int..

[45] Sergeeva N.S., Kanukoev K.Y., Karmakova T.A., Alentov I.I., Marshutina N.V., Kaprin A.D. (2021). On normalizing of urinary KIM-1 level to urine creatinine in patients with renal cell cancer. Klin. Lab. Diagn.

[46] Murray P.T., Mehta R.L., Shaw A., Ronco C., Endre Z., Kellum J.A., Chawla L.S., Cruz D., Ince C., Okusa M.D. (2014). ADQI 10 workgroup. Potential use of biomarkers in acute kidney injury: Report and summary of recommendations from the 10th Acute Dialysis Quality Initiative consensus conference. Kidney Int..

[47] Booke H., von Groote T., Zarbock A. (2024). Ten tips on how to reduce iatrogenic acute kidney injury. Clin. Kidney J..

