# Supplementary material for: Tubular Damage Biomarkers Are a Useful Tool for Identifying Early Renal Injury in Long COVID
Source: Int J Mol Sci. 2026 Mar 6;27(5):2420. doi: 10.3390/ijms27052420 (PMC12985994; doi:10.3390/ijms27052420)
Supplement: Supplementary file 1 [file ijms-27-02420-s001.zip › ijms-4140493-supplementary.pdf]

Supplementary Materials

Tubular damage biomarkers are a useful tool for identifying early renal injury in Long COVID

Table of contents

Supplementary Materials	Page #
**Supplementary Methods**	**3-5**
**Supplementary Tables**	**5**
**Supplementary Table S1.** Regular medication used prior to SARS-CoV2 infection.	**5**
**Supplementary Table S2.** Vaccination rate and respiratory function at acute COVID-19 hospitalization.	**6**
**Supplementary Table S3.** Therapeutic interventions during acute COVID-19 hospitalization and associated outcomes.	**7**
**Supplementary Table S4.** Laboratory findings and estimated glomerular filtration rate (eGFR) at the time of urine collection.	**8**
**Supplementary Table S5.** Patient flow diagram: enrollment, screening, eligibility criteria and groups analyzed.	**9**
**Supplementary References**	**10**

Supplementary Methods

Research and Data Acquisition Protocol

During the visit to Clementino Fraga Filho University Hospital (HUCFF), each patient was directed to a private room, where the objectives of the study were explained, along with a thorough explanation of the patients' rights and guarantees of privacy. After confirming their willingness to participate, the Informed Consent Form was signed.

The patient was then referred to the Long COVID outpatient clinic, where a clinical consultation was conducted by a pulmonology specialist. During this consultation, data were collected through anamnesis and physical examination, enabling the acquisition of clinical and demographic information such as sex, age, body mass index (BMI), and presence of comorbidities. Additionally, the patients' electronic medical records were reviewed for retrospective extraction of clinical data recorded during hospitalization for COVID-19, including the clinical spectrum of symptoms used to classify infection severity [24]. Information was also collected regarding prior medication use, laboratory test results, therapeutic interventions, clinical outcomes, complications, ICU admission, and vaccination status at the time of hospitalization.

Subsequently, patients were scheduled to return to the hospital for blood and urine sample collection. On the morning of collection, spot urine and blood samples were obtained by a nurse. Urine samples were collected for the evaluation of urinary levels of creatinine, protein, albumin, β2-microglobulin (Uβ2M), and kidney injury molecule 1 (UKIM1). Blood samples were sent to the HUCFF Clinical Analysis Laboratory, where standard protocols were used to assess the following parameters: blood count, C-reactive protein (CRP), creatinine, lactate dehydrogenase (LDH), alanine aminotransferase (ALT), ferritin, creatine phosphokinase (CPK), and D-dimer.

This study does not include a placebo group, intervention procedures, or randomization.

Classifications used in the study

The clinical spectrum of SARS-CoV-2 infection was defined according to the criteria established by the National Institutes of Health (NIH), which outlined four categories based on the recommendations of the World Health Organization (WHO) for staging COVID-19, as published in the *Coronavirus Disease 2019 (COVID-19) Treatment Guidelines* [24].

Patients with mild disease presented with classic respiratory symptoms caused by COVID-19, but without hypoxemia, dyspnea, or pulmonary abnormalities on imaging. Moderate disease was defined by evidence of lower respiratory tract involvement identified through clinical examination or imaging, without impairment of peripheral oxygen saturation (SpO_2 >94%). Severe disease was characterized by SpO_2 <94% on room air, a partial pressure of arterial oxygen to inspired oxygen fraction ratio (PaO_2/FiO_2) <300 mmHg, respiratory rate >30 breaths per minute, or pulmonary infiltrates on chest computed tomography (CT) involving more than 50% of the lung fields. Critical disease, in turn, was defined in patients who developed respiratory failure, septic shock, or signs of organ dysfunction.

Analysis of Urinary Samples

Urinary protein and creatinine concentrations were measured using colorimetric methods with the Sensiprot and Creatinine K kits (Labtest, Lagoa Santa, MG, Brazil). Urinary albumin was quantified using a turbidimetric method with the Urine/CSF Albumin kit (#B38858, Beckman Coulter UA, Brea, CA, USA). UKIM1 levels were determined by ELISA using the Human HAVCR1 kit (#EHHAVCR1, Thermo Fisher

Scientific, Waltham, MA, USA), and Uβ2M was measured using an ELISA kit (#ab181423, Abcam, Cambridge, MA, USA). All assays were performed according to the manufacturers' instructions.

Supplementary Tables

Supplementary Table S1. Regular medication used prior to SARS-CoV2 infection.

	No	All patients	6 months post COVID-19 (6-MPC)	12 months post COVID-19 (12-MPC)	24 months post COVID-19 (24-MPC)	*P* value between groups*
**Absolute and relative frequencies, *n* (%)**		**75**	**36 (48%)**	**24 (32%)**	**15 (20%)**	
**Use of previous medication, *n* (%)**	75	65 (86.7%)	30 (83.3%)	21 (87.5%)	14 (93.3%)	0.752
ACEi/ARB	75	40 (53.3%)	15 (41.7%)	15 (62.5%)	10 (66.7%)	0.155
Diuretics	75	16 (21.3%)	5 (13.9%)	8 (33.3%)	3 (20%)	0.196
Beta-blocker	75	15 (20%)	8 (22.2%)	5 (20.8%)	2 (13.3%)	0.764
Oral hypoglycemic	75	19 (25.3%)	6 (16.7%)	9 (37.5%)	4 (26.7%)	0.190
Insulin	75	8 (10.7%)	3 (8.3%)	4 (16.7%)	1 (6.7%)	0.506
Statin	75	28 (37.3%)	10 (27.8%)	13 (54.2%)	5 (33.3%)	0.110
Anticoagulant	75	3 (4%)	2 (5.6%)	1 (4.2%)	0 (0%)	0.653
Antiplatelet	75	11 (14.7%)	3 (8.3%)	4 (16.7%)	4 (26.7%)	0.228
Vitamin D	75	9 (12%)	5 (13.9%)	2 (8.3%)	2 (13.3%)	0.798
Bronchodilator	75	5 (6.7%)	1 (2.8%)	3 (12.5%)	1 (6.7%)	0.335
Other immunosuppressants	75	9 (12%)	5 (13.9%)	3 (12.5%)	1 (6.7%)	0.767

Legend: The descriptive analysis of the data is presented using absolute frequencies (*n*) and percentages according to group. No = Number of observations; *Statistical significance was determined using the tests described in the statistical analysis section. A $p < 0.05$ was considered statistically significant.

Supplementary Table S2. Vaccination rate and respiratory function at acute COVID-19 hospitalization.

	No	All patients	6 months post COVID-19 (6-MPC)	12 months post COVID-19 (12-MPC)	24 months post COVID-19 (24-MPC)	*P* value between groups*
**Absolute and relative frequencies, *n* (%)**		**75**	**36 (48%)**	**24 (32%)**	**15 (20%)**	
**Vaccinated with at least 1 dose, *n* (%)**	75	39 (52%)	31 (86.1%)	8 (33.3%)	1 (6.7%)	<0.001
**Hospitalization laboratory parameters**						
SpO_2, M [SD], %	64	93.8 [6.1]	97 [2.4]	92 [5.4]	90.2 [9.1]	<0.001
Respiratory Rate, M [SD], irpm	54	22.4 [7.9]	17.1 [5.9]	24.5 [6.9]	26.4 [8.3]	<0.001
Hemoglobin, M [SD], g/dL	75	10.2 [3.2]	9.8 [3.5]	11.2 [2.4]	9.8 [3.4]	0.206
Lymphocytes, MD [Q1-Q3], (/mm^3)	75	720 [505-1190]	1140 [613.8-1896]	526 [377-742]	693 [524-821]	<0.001
Platelets, MD [Q1-Q3], (10^3/mm^3)	75	154000 [288-236500]	179000 [335.2-263750]	139000 [172.5-226500]	146000 [10647-185000]	0.479
PCR, MD [Q1-Q3], mg/L	72	129.9 [31.23-220.4]	46 [14.7-129.7]	191.1 [110.2-277.2]	156 [106.3-307.1]	<0.001
Creatinine, MD [Q1-Q3], mg/dL	75	1 [0.8-1.4]	0.9 [0.7-1.3]	1 [0.9-1.3]	1.1 [0.9-1.6]	0.161
LDH, M [SD], U/L	45	483.93 [370.2]	276.2 [182.7]	508.8 [257.5]	604.9 [520.9]	0.031
ALT, MD [Q1-Q3], U/L	62	53 [21-109.5]	25 [17.5-47]	75.5 [36.2-117]	76 [59-133.5]	0.008
Ferritin, MD [Q1-Q3], ng/mL	40	1036.5 [473.8-2595]	406 [191.5-865.8]	1069 [633-2178]	1424 [739-3018.5]	0.067
CPK, MD [Q1-Q3], U/L	37	131 [54-361]	86 [50.2-873.2]	70 [40-260]	202.5 [105.5-388.5]	0.301
D-dimer, MD [Q1-Q3], ng/mL	18	1234 [900-2429]	989.5 [626.2-4024]	1435 [1013.2-2584]	1253 [626.2-1879]	0.698

Legend: The descriptive analysis of the data is presented using absolute frequencies (*n*) and percentages according to group. No = Number of observations; M = Mean; MD = Median; SD = Standard Deviation; Q1 = First Quartile; Q3 = Third Quartile; *Statistical significance was determined using the tests described in the statistical analysis section. A $p < 0.05$ was considered statistically significant.

Supplementary Table S3. Therapeutic interventions during acute COVID-19 hospitalization and associated outcomes.

	No	All patients	6 months post COVID-19 (6-MPC)	12 months post COVID-19 (12-MPC)	24 months post COVID-19 (24-MPC)	*P* value between groups*
**Absolute and relative frequencies, *n* (%)**		**75**	**36 (48%)**	**24 (32%)**	**15 (20%)**	
**Treatment during hospitalization, *n* (%)**						
Corticosteroid	75	35 (46.7%)	8 (22.2%)	23 (95.8%)	4 (26.7%)	<0,001
Inhaled corticosteroid	75	4 (5.3%)	0 (0%)	3 (12.5%)	1 (6.7%)	0.081
Oral corticosteroid	75	7 (9.3%)	5 (13.9%)	2 (8.3%)	0 (0%)	0.393
O2 supplementation	75	38 (50.7%)	4 (11.1%)	21 (87.5%)	13 (86.7%)	<0.001
Mechanical ventilation	74	9 (12.2%)	1 (2.9%)	4 (16.7%)	4 (26.7%)	0.036
Non-invasive ventilation	75	3 (4%)	0 (0%)	3 (12.5%)	0 (0%)	0.037
**ICU admission, *n* (%)**	75	25 (33.3%)	4 (11.1%)	17 (70.8%)	4 (26.7%)	<0.001
**Complications, *n* (%)**	75	18 (24%)	6 (16.7%)	6 (25%)	6 (40%)	0.204
Deep vein thrombosis	75	3 (4%)	0 (0%)	1 (4.2%)	2 (13.3%)	0.074
Pulmonary thromboembolism	74	1 (1.4%)	0 (0%)	1 (4.2%)	0 (0%)	0.514
Arrhythmia	75	4 (5.3%)	1 (2.8%)	1 (4.2%)	2 (13.3%)	0.319
Neurological	75	2 (2.7%)	1 (2.8%)	1 (4.2%)	0 (0%)	1.000
Acute kidney injury	75	4 (5.3%)	0 (0%)	1 (4.2%)	3 (20%)	0.019
Acute coronary syndrome	75	2 (2.7%)	1 (2.8%)	0 (0%)	1 (6.7%)	0.462
Others	74	10 (13.3%)	5 (13.9%)	4 (16.7%)	1 (6.7%)	0.824

Legend: The descriptive analysis of the data is presented using absolute frequencies (*n*) and percentages by group. No = Number of observations; *Statistical significance was determined by the tests described in the statistical analysis section. A $p < 0.05$ was considered statistically significant.

Supplementary Table S4. Laboratory findings and estimated glomerular flow rate (eGFR) at the time of urine collection.

	No	All patients	6 months post COVID-19 (6-MPC)	12 months post COVID-19 (12-MPC)	24 months post COVID-19 (24-MPC)	*P* value between groups*
**Absolute and relative frequencies, *n* (%)**		**75**	**36 (48%)**	**24 (32%)**	**15 (20%)**	
**Follow-up laboratory parameters**						
Hemoglobin, MD [Q1-Q3], g/dL	73	13.2 [11.8-14.2]	12.3 [10.9-13.8]	13.8 [13-14.5]	13.8 [12.5-14.6]	0.017
Lymphocytes, MD [Q1-Q3], (/mm^3)	73	1967 [1340-2650]	2000 [1320-2700]	1930 [1472.5-2800]	2029 [1632.5-2208]	0.835
Platelets, MD [Q1-Q3], (10^3/mm^3)	73	243000 [187000-311000]	275000 [224000-332000]	232500 [179250-300000]	244500 [179000-289500]	0.121
PCR, MD [Q1-Q3], mg/L	68	3.4 [2.1-7.5]	4.2 [2.3-10.9]	3.4 [2.8-7]	2.3 [1.5-3.6]	0.144
Creatinine, MD [Q1-Q3], mg/dL	74	0.9 [0.7-1]	0.8 [0.7-1]	0.9 [0.78-1]	0.9 [0.8-1.1]	0.512
LDH, MD [Q1-Q3], U/L	72	188.5 [155-214]	185 [149-214]	181.5 [165-209.5]	197 [185.5-220.5]	0.580
ALT, MD [Q1-Q3], U/L	64	16 [13-28]	14.5 [11.75-28.3]	18 [15-24.5]	24 [16-28]	0.103
Ferritin, MD [Q1-Q3], ng/mL	68	123 [62.5-263.5]	102.5 [48.3-229.5]	160 [93-240]	130 [94.5-362]	0.318
CPK, MD [Q1-Q3], U/L	62	84 [59.8-121.5]	67.5 [50.75-120.3]	95 [69-108]	95.5 [79.8-156]	0.076
D-dimer, MD [Q1-Q3], ng/mL	66	632 [437.3-1363.5]	694.5 [507.5-2046.8]	599.5 [404.8-963.3]	624 [389.5-913]	0.144

Legend: The descriptive analysis of the data is presented using absolute frequencies (*n*) and percentages according to group. No = Number of observations; M = Mean; MD = Median; SD = Standard Deviation; Q1 = First Quartile; Q3 = Third Quartile; *Statistical significance was determined using the tests described in the statistical analysis section. A $p < 0.05$ was considered statistically significant.

Supplementary Table S5. Patient flow diagram: enrollment, screening, eligibility criteria, and groups analyzed.

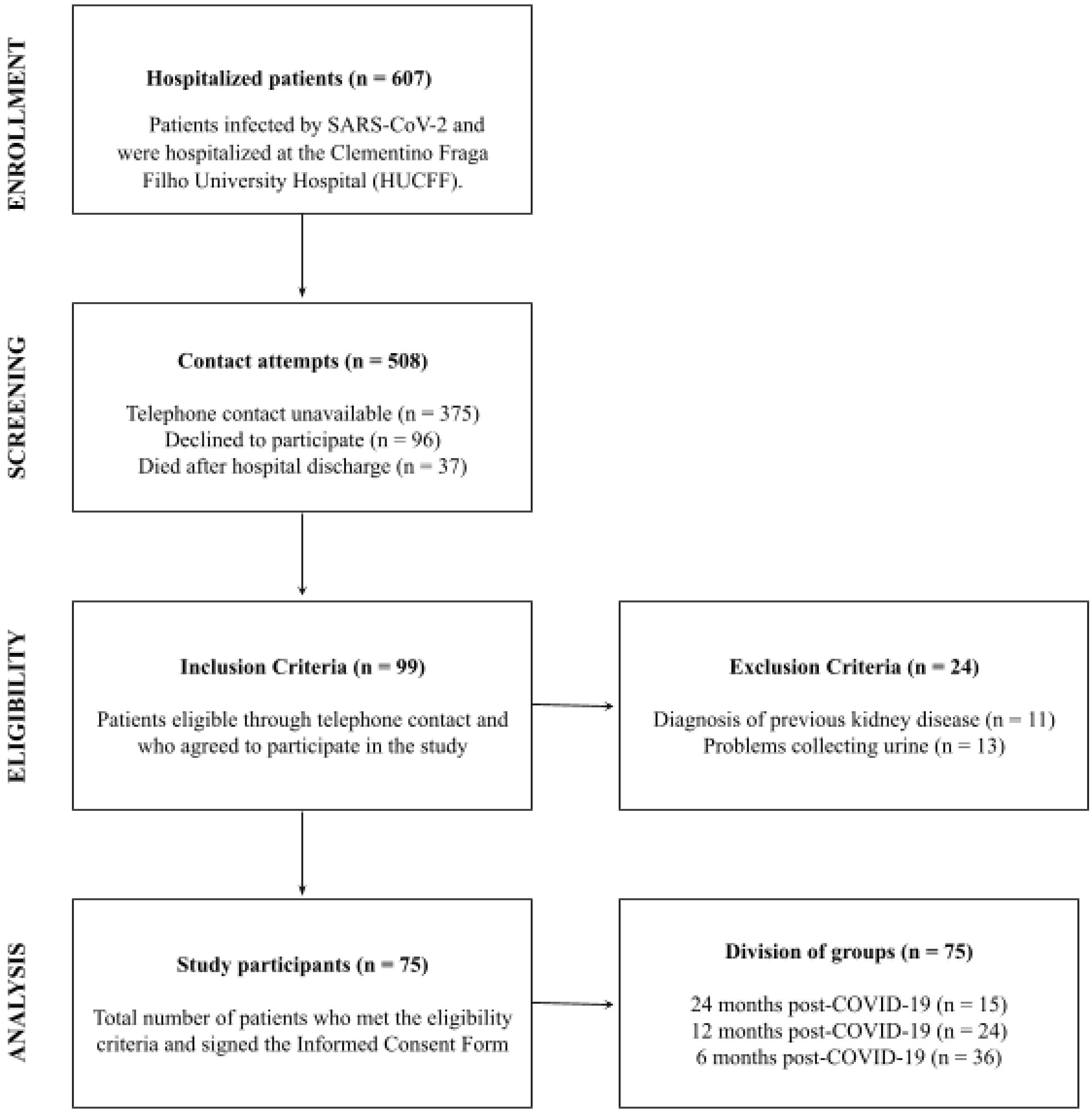

Supplementary References

24. National Institutes of Health (US). COVID-19 Treatment Guidelines Panel. Coronavirus Disease 2019 (COVID-19) Treatment Guidelines. Available online: https://www.ncbi.nlm.nih.gov/books/NBK570371/pdf/Bookshelf_NBK570371.pdf (accessed on 26 May 2025).
